# MicroRNA-769-3p inhibits tumor progression in glioma by suppressing ZEB2 and inhibiting the Wnt/β-catenin signaling pathway

**DOI:** 10.3892/ol.2021.12489

**Published:** 2021-01-26

**Authors:** Kai Wang, Shasha Yang, Yishen Gao, Caihong Zhang, Qiangbo Sui

Oncol Lett 19: 992-1000, 2020; DOI: 10.3892/ol.2019.11135

Following the publication of the above paper, an interested reader drew to the authors’ attention that there were inconsistencies in the description of the results shown in Fig. 2B, comparing between the Results and the Discussion sections.

Upon investigating this matter with the authors, the authors have realized that the description of the results concerning this experiment was correct in the Discussion, but was wrong in the Results section (and elsewhere). First, Fig. 2B was labelled incorrectly, and the corrected version of Fig. 2 is shown oppossite. Otherwise, the text relating to this Figure should have been written as follows (changes are highlighted in bold):

(i) Abstract, p. 992, line 21: “**Low** tissue miR-769-3p expression predicted poor overall survival in patients with glioma (log-rank P=0.001)”;

(ii) Results section, p, 994, *Prognostic value of tissue miR-769-3p level in patients with glioma* subsection, line 4: “The results demonstrated that patients with a **low** miR-769-3p expression in the glioma tissue exhibited relatively poor overall survival compared with those in the high expression group (log-rank P=0.001; Fig. 2B).”

(iii) Fig. 2B legend, p. 996: “(B) Patients with **low** miR-769-3p expression exhibited shorter survival times compared with those with **high** miR-769-3p expression.”

The authors are grateful to the Editor of *Oncology Letters* for allowing them the opportunity to publish this Corrigendum; furthermore, the authors apologize for any inconvenience caused to the readership of the Journal.

## Figures and Tables

**Figure 2.**
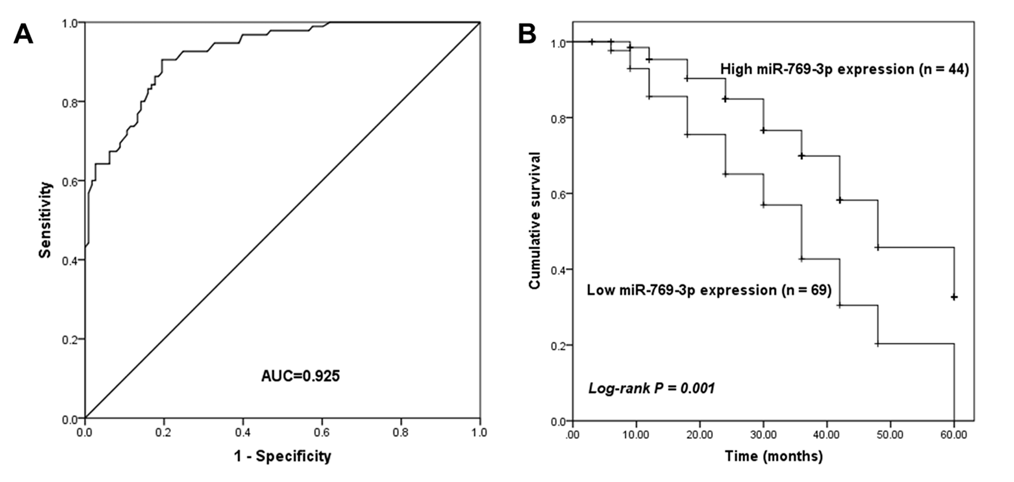
Diagnostic value of miR-769-3p in glioma. (A) The receiver operating characteristic curve analysis of patients with glioma based on the serum miR-769-3p levels demonstrated an AUC value of 0.925, with the sensitivity and specificity of 90.5 and 80.5%, respectively. (B) Survival analysis in patients with glioma based on the tissue expression of miR-769-3p. Patients with **low** miR-769-3p expression exhibited shorter survival times compared with those with **high** miR-769-3p expression. AUC, area under the curve; miR, microRNA.

